# Impact of annual and semi-annual mass drug administration for Lymphatic Filariasis and Onchocerciasis on Hookworm Infection in Côte d’Ivoire

**DOI:** 10.1371/journal.pntd.0008642

**Published:** 2020-09-25

**Authors:** Agodio Loukouri, Aboulaye Méité, Benjamin G. Koudou, Charles W. Goss, Daphne Lew, Gary J. Weil, Eliezer K. N’Goran, Peter U. Fischer

**Affiliations:** 1 Laboratoire de Zoologie et Biologie Animale, UFR Biosciences, Université Félix Houphouët-Boigny, Abidjan, Côte d'Ivoire; 2 Programme National de Lutte contre les Maladies Tropicales Négligées à Chimiothérapie Préventive, Abidjan, Côte d’Ivoire; 3 Centre Suisse de Recherches Scientifiques en Côte d’Ivoire, Abidjan, Abidjan, Côte d’Ivoire; 4 Laboratoire de Cytologie et Biologie Animale, UFR Science de la Nature, Université Nangui Abrogoua Abidjan, Abidjan, Côte d’Ivoire; 5 Division of Biostatistics, Washington University School of Medicine, St. Louis, Missouri, United States of America; 6 Infectious Diseases Division, Department of Medicine, Washington University School of Medicine, St. Louis, Missouri, United States of America; Imperial College London, Faculty of Medicine, School of Public Health, UNITED KINGDOM

## Abstract

Mass Drug Administration (MDA) programs to eliminate Lymphatic Filariasis (LF) in western Africa use the anthelminthics ivermectin plus albendazole. These drugs have the potential to impact also Soil-Transmitted Helminth (STH) infections, since the drugs have a broad range of anthelminthic activity. Integration of preventive chemotherapy efforts for LF, onchocerciasis and STH is recommended by the World Health Organization (WHO) in order to avoid duplication of MDA and to reduce costs. The objective of the current study was to determine whether five semi-annual rounds of community-wide MDA to eliminate LF and onchocerciasis have a greater impact on STH than three annual rounds of MDA with similar compliance. The effects of MDA using ivermectin (IVM, 0.2 mg/kg) combined with albendazole (ALB, 400 mg) on the prevalence and intensity of hookworm infection were evaluated in the Abengourou (annual MDA) and Akoupé (semi-annual MDA) health Districts in eastern Côte d’Ivoire from 2014 to 2017. A cross-sectional approach was used together with mixed logistic regression, and mixed linear models. Subjects were tested for STH using the Kato-Katz technique before the first round of MDA and 12, 24, and 36 months after the first round of MDA. The mean self-reported MDA compliance assessed during the survey was 65%, and no difference was observed between treatment areas. These results were confirmed by an independent coverage survey as recommended by WHO. Hookworm was the most prevalent STH species in both areas (23.9% vs 12.4%) and the prevalence of other STH species was less than 1%. The crude prevalence of hookworm dropped significantly, from 23.9% to 5.5% (*p* <0.001, 77% reduction) in the annual MDA treatment area and from 12.4% to 1.9% (*p* <0.001, 85% reduction) in the semi-annual treatment area. The average intensity of hookworm infection decreased in the annual MDA area (406.2 epg to 118.3 epg), but not in the semi-annual MDA area (804.9 epg to 875.0 epg). Moderate and heavy infections (1% and 1.3% at baseline) were reduced to 0% and 0.4% in the annual and semi-annual treatment areas, respectively. Using a mixed logistic regression model, and after adjusting for baseline prevalence, only the year 2 re-examination showed a difference in prevalence between treatments (OR: 2.26 [95% CI: 1.03, 4.98], *p* = 0.043). Analysis of intensity of hookworm infection indicated also that treatment differences varied by follow-up visit. In conclusion twelve months after the last treatment cycle, three annual and five semi-annual rounds of community-wide MDA with the combination of IVM and ALB showed strong, but similar impact on hookworm prevalence and intensity in eastern Côte d’Ivoire. Therefore, an annual MDA regimen seems to be an efficient strategy to control hookworm infection in endemic areas with low and moderate infection prevalence.

**Trial registration**: The study was registered at ClinicalTrial.gov under the number NTC02032043.

## Author summary

Community-wide MDA to eliminate LF and onchocerciasis has the beneficial effect to reduce also STH infections. The objective of the current study was to determine whether five semi-annual rounds of MDA have a greater impact on STH than three annual rounds of MDA using ivermectin combined with albendazole. In Abengourou and Akoupé health Districts in eastern Côte d’Ivoire the prevalence and intensity of hookworm infection were evaluated before and after MDA. Prior to MDA and after each annual treatment cycle, study participants were tested for STH using the Kato-Katz technique. The mean MDA compliance assessed during the survey was 65%, and no difference was observed between treatment areas. Compliance results were confirmed by an independent coverage survey as recommended by WHO. Hookworm was the most prevalent STH species in both areas and the prevalence of other STH species was less than 1%. The crude prevalence of hookworm dropped significantly, from 23.9% to 5.5% in the annual and from 12.4% to 1.9% in the semi-annual treatment areas. The intensity of hookworm infection in infected persons decreased significantly in the annual MDA area (406.2 epg to 118.3 epg, *p* = 0.017), but not in the semi-annual MDA area (804.9 epg to 875.0 epg, *p =* 0.216). Moderate and heavy infections were reduced to less than 1% in both treatment areas. Three annual and five semi-annual rounds of community-wide MDA with the combination of IVM and ALB showed strong, but similar effects on hookworm prevalence and intensity in eastern Côte d’Ivoire. Therefore, an annual MDA regimen seems to be an efficient strategy for controlling hookworm infection in endemic areas with low and moderate infection prevalence.

## Introduction

Soil-Transmitted Helminth (STH) infections are one of the most common Neglected Tropical Diseases (NTDs) and cause significant public health problems in many poor communities located in tropical and sub-tropical countries [1]. Approximately 1.5 billion people are infected with at least one STH species [2], and more than 300 million show clinical signs including: pneumonitis referred to as Loeffler’s syndrome [3], intestinal obstruction [4], anemia [5], abdominal colitis and pain, diarrhea, dysentery associated with rectal prolapse, malnutrition, stunted growth [6] and long-term disability or early death.

Resolutions of the World Health Organization (WHO) call for intensified control of STH, and this is supported by pharmaceutical companies, international aid agencies and philanthropic organizations [7, 8]. The WHO focuses on the most vulnerable populations such as pre-school children, school-aged children and women of child-bearing age. It calls for regular deworming to avert widespread morbidity and reduce the occurrence, extent, severity and long-term consequences of morbidity, and reduce transmission [9]. Twice yearly Mass Drug Administration (MDA) of the target population using the anthelminthics albendazole (ALB) or mebendazole (MEB) is recommended in high risk communities (prevalence ≥50%) and annual MDA is recommended for low risk communities (20% ≤ prevalence <50%) [7]. Unlike for the elimination of lymphatic filariasis (LF) and onchocerciasis WHO does not recommend community-wide MDA for STH at present.

Large parts of Sub-Saharan Africa are not only endemic for STH but also for LF and onchocerciasis. A key intervention strategy to eliminate LF and onchocerciasis is MDA using ivermectin (IVM) alone (for onchocerciasis) or IVM combined with ALB (for LF). This strategy is directed at the entire population at risk of infection aged 5 years or older [10]. Both drugs have a broad range of anthelminthic activity and are also effective against STH [11, 12]. The combination of IVM and ALB has higher cure and egg reduction rates for STH compared to a single dose of either ALB, MEB or IVM [13]. MDA using IVM plus ALB to the entire community reduce STH transmission, because of its effect on the parasite reservoir. This is especially true for hookworm, because adults serve as a major reservoir for that infection [14]. Twice-yearly community MDA might lead to further reductions in parasite intensity and infection prevalence. This will support the ambitious goal of WHO to control morbidity, and eventually break transmission of STH [8]. Integration of STH intervention with LF and/or onchocerciasis elimination programs prevents unnecessary duplication of MDA, thus reducing costs [7, 15].

Like other countries in West Africa, Côte d’Ivoire is endemic for STH [16–18]. Since the early 2000s school-based deworming for STH was linked with the polio vaccination campaign, the vitamin A distribution program, the World Food Program (WFP) [8], and in selected areas with the Schistosomiasis Consortium for Operational Research and Evaluation (SCORE) project [19]. The MDA scheduled for school-aged children varied based on the epidemiological status in the target population and the availability of financial resources. Although specific funds were available for community-wide MDAs to treat schistosomiasis, onchocerciasis and LF, no specific funds for STH control were available in Côte d’Ivoire during our study. Therefore, in areas where STH are not co-endemic with onchocerciasis or LF, no MDA for the control of STH was performed.

The health Districts of Abengourou and Akoupé, in the eastern Departments of Côte d’Ivoire are co-endemic for LF, onchocerciasis and STH. Prior to 2009, several rounds of annual IVM (Community Directed Treatment with IVM, CDTi) were provided for onchocerciasis control to smaller villages (≤ 3000 residents) within a radius of 5 miles of a river. Abengourou and Akoupé health Districts were targeted by the national LF elimination program to receive MDA using IVM plus ALB. This MDA was initiated in 2014 in parallel with the start of the present study. This study compared the impact of annual (3 rounds) and semi-annual MDA (5 rounds) on hookworm, the most prevalent STH species. We used a cross-sectional approach (S1 Strobe checklist) to assess differences in drug coverage and hookworm intensity and prevalence.

## Methods

### Ethical approval and selection of study areas

This study received ethical clearance from institutional review boards at Washington University School of Medicine (St. Louis, MO) and at the National Committee of Ethics and Research of Côte d’Ivoire (CNER/797/Ti/15). The study was registered at ClinicalTrial.gov under the number NTC02032043. The administrative and village authorities were informed about the procedures, benefits and potential risks of the project. Oral informed consent from each participant in the study was obtained and documented in the electronic data capture system. Both institutional review boards recognized that oral informed consent for this kind of study is in agreement with the local traditions in the study communities. Inclusion of minors in the study required consent of one parent and assent of the child. The confidentiality of the data was ensured through a coding process, and the project datasets did not contain personal identifying information.

The health Districts of Akoupé and Abengourou were co-endemic for LF, onchocerciasis and STH and eligible for community-wide MDA with IVM plus ALB. Study villages had a baseline *Wuchereria bancrofti* microfilaremia rate of at least 10% and an *Onchocerca volvulus* nodule rate of at least 25%. Furthermore, villages eligible for the study had no previous MDA for at least 12 months, were accessible by four-wheel drive cars, and expressed willingness to participate in the community surveys. Among these eligible villages, four per health district were randomly selected for inclusion in this cross-sectional study (S1 Table).

### Study areas and population

**Fig 1:**
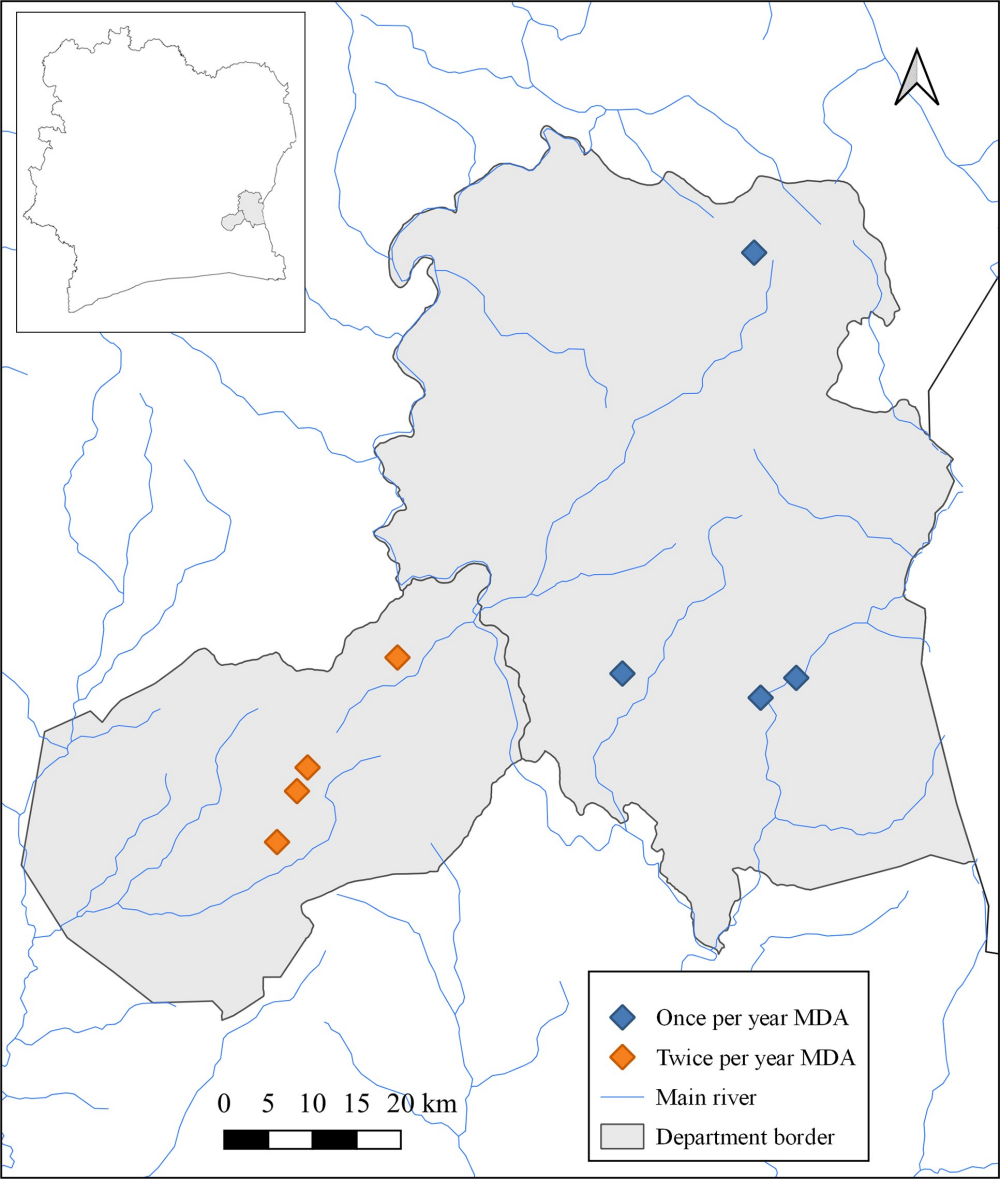
Map of study sites in the health Districts of Abengourou and Akoupé in eastern Côte d’Ivoire. Geographic coordinates of each study site were recorded using a hand-held global positioning system (GPS) receiver of the cell phone and Arc Map 10.2.1 (Environmental Systems Research Institute Inc.; Redlands, USA) was used to generate the map of the study sites.

The study areas are located in the forest zone of eastern Côte d’Ivoire (Fig 1, S1 Table). The areas are characterized by a warm and humid climate. Study villages were rural agricultural communities. The total population of Akoupé and Abengourou health Districts (sub-prefectures) is 80,000 and 175,000, respectively and the population size of the study villages ranged between 500 and 3,200 residents. Anthelminthic drugs were available at local markets and at District hospital pharmacy. Study participants from both districts reported similar access to running water and latrines. However, people from Akoupé mentioned that they travelled more frequently to the Abidjan metropolitan area. We have no evidence that the living condition have changed during the study period. Eligible for the study were individuals aged five years or above and without any evidence of acute illness or severe chronic disease. Pregnant women were not eligible to receive MDA. No school-based deworming was performed during the study period.

### Size of study population

The sample size was calculated for a two-sided test with a power of 80%, and an α-level of 0.05. We estimated that prevalence of STH was around 20%. Thus, a total sample of at least 1,600 stools samples (800 per area) was required for impact assessment.

### Demographics

Personal information such as name, sex, age was recorded in the electronic data capture system and linked to the barcode. Study participants were examined for LF, onchocerciasis and a subset for STH in parallel. All laboratory samples were labelled by barcode only and results per barcode were entered in a specific module. Later, the complete data set was linked and stripped from personal identifying information before analysis.

### Parasitological examination

STH testing was performed at baseline and at one year intervals after MDA. The last STH survey was performed one year after the last round of MDA in both treatment areas. In each study area 1,200 stool containers (800 stool container + 50%) per area were distributed per year to potential study participants. Since about 2,000 subjects were examined for LF and onchocerciasis, three out of five enrolled subjects received a stool container. Stool containers were labeled with a barcode for each participant. Sometimes containers were also marked with a sign or initials if many children of a family received a container to reduce the risk of a mix-up. During the survey, subjects were instructed to produce stool on a piece of paper and transfer a walnut size piece into the container. The containers were collected during the day or at the latest at the following morning, kept cool and transported to the field laboratory for immediate examination. Stool samples were tested with duplicate Kato-Katz smears on the same day of collection [20]. Slides prepared from the same specimen were labeled with the patient ID (barcode) and read within 60 min after preparation. Each of the two slides was examined by one of the two microscopists using 40x magnification. Quality control of the slides was carried out by a third experienced parasitologist. Ten percent of the slides were randomly selected and reexamined. In cases of major disagreement between reading results, the entire series of slides with differing results was reexamined. Reading results of the slides were recorded on a paper form containing the barcode and results were subsequently entered into an electronic data capture system. Analysis of 25 hookworm egg positive stool samples by qPCR [21], revealed that all samples were positive for *Necator americanus* and negative for *Ancylostoma duodenale*.

### Mass drug administration using ivermectin plus albendazole

The MDA was performed by the National Program for the Elimination of NTDs. Treatments were initiated in 2014 and performed at month 0, 6,12, 18 and 24 (in Akoupé health District, semi-annual MDA, 5 treatment rounds) and month 0, 12 and 24 (Abengourou health District, annual MDA, 3 treatment rounds). Training sessions were held for health workers of the different study sites who assisted the national team. Local health workers were trained to administer the drugs and detect, manage and refer when necessary, adverse events following treatment. Eligible inhabitants received directly observed oral treatment with IVM (0.2 mg/kg) and ALB (a fixed dose of 400 mg). For dosing of IVM a dosing pole was used [22]. As recommended by WHO, an independent coverage survey was conducted in both Districts in 2017 by an experienced social scientist [23].

### Assessment of compliance to treatment

Data on compliance to treatment was recorded during the cross-sectional surveys, 12, 24 and 36 months after the start of MDA. The reported compliance was calculated on the base of the proportion of the study population who reported during the follow-up to have swallowed once (annual treatment area), or twice (semi-annual treatment area) in the previous year. In addition, an independent coverage survey according to WHO guidelines was conducted in Akoupé about 3 months after the last follow-up survey/treatment in 2017 [24].

### Data management and analysis

For the baseline survey and the first follow-up, demographic and laboratory data were recorded and synchronized using Motorola cellular telephones (XT 720; Motorola, Chicago, IL) before being sent to the server using the LinksSystem [19]. For later surveys, data were collected using EpiInfo for Mobile Devices App (Centers for Disease Control and Prevention, Atlanta, GA); encrypted data were transferred to an Azure cloud server (Microsoft, Seattle WA). For analysis, the data were downloaded as Excel files from the servers. Study participants were classified into six age groups: ≤10 years, 11–20 years, 21–30 years, 31–40 years, 41–50 years, ≥ 51 years. Intensity of infection was expressed as eggs per gram (epg). The mean infection intensity was computed using the arithmetic mean of infected subjects. Infection intensity was stratified into three categories (light, moderate and heavy) according to WHO thresholds [25]. *A*. *lumbricoides* was defined between 1 epg to 4999 epg, 5000 epg to 49999 epg, and ≥50000 epg. The species *T*. *trichiura* was defined as follows: 1 epg to 999 epg, 1000 epg to 9999 epg, and ≥ 10000 epg. *Ancylostoma* spp. eggs were grouped between 1 epg and 1999 epg, 2000 epg and 3999 epg, and ≥4000 epg. *S*. *mansoni* was as follows: 1 epg to 99 epg, 100 epg to 399 epg, and ≥400 epg. Cross-sectional data were used to evaluate changes in infection rates and intensities in study areas. Differences in infection rates were assessed by Chi-squared tests. Change in infection intensity were assessed using Mann Whitney U tests.

In order to better compare results from the two Districts, mixed-effects logistic regression models and mixed linear models were used to analyze hookworm presence and intensity, respectively. Our statistical models included variables that corresponded to MDA treatment (annual vs semi-annual), visit (baseline, year 1, year 2, year 3) and their interaction. Village was treated as a random effect to account for intra-cluster correlation. All models were adjusted for age and sex because these covariates are thought to be associated with hookworm infection. To account for infection prevalence differences between treatment groups at baseline, we conducted an additional analysis where we analyzed follow-up data only (year 1, year 2, year 3) and adjusted for baseline prevalence in each village. We imputed mean prevalence values for villages in each treatment area that were not included in the baseline sample. *p*-values < 0.05 were considered significant. Statistical analyses were conducted using STATA 12.00 software (Stata Corp., Texas, USA) and SAS version 9.4 (SAS Institute Inc., NC, USA).

## Results

### Demographics and treatment reported compliance

For the assessment of STH we recruited 2,022 individuals at baseline (1,030 in the annual MDA area and 992 in the semi-annual MDA area), 1,911 at the first follow up (1,112 vs 799, respectively), 1,431 at the second follow up (748 vs 683, respectively), and 1,161 at the third follow up (531 vs 630, respectively). Between 48 and 84% of the subjects who received a stool container provided a stool sample suitable for analysis. The mean age of study participants in the baseline survey was 31.2 years and 29.1 years for annual and semi-annual treatment areas, respectively. The gender ratio (males per female) was 1.0 and 0.7, in annual and semi-annual areas. The rate of compliance with treatment was assessed during the survey 6 to 12 months after the last MDA. The mean of reported compliance was 65.2% in annual MDA area and 64.3% in semi-annual MDA area, with a range from 63.9% to 67.3% and from 62.1% to 66.2%, respectively, over the period of study. However, no statistically significant difference was observed between treatment areas at follow up 1 (p = 0.267), follow up 2 (p = 0.762), and follow up 3 (p = 0.400). An independent compliance survey according to WHO guidelines in 2017 confirmed the results and found a mean rate of compliance of 71.3% in the semi-annual MDA area.

### Prevalence of soil-transmitted helminths

During the baseline survey (pre-MDA) eggs of four helminth species (*A*. *lumbricoides*, hookworm [*N*. *americanus*], *T*. *trichiura* and *S*. *mansoni*) were found in stool samples from Abengourou (annual MDA) and Akoupé (semi-annual MDA). At baseline the hookworm prevalence was two times higher in the annual MDA area (23.9%) than in the semi-annual area (12.4%). Prevalence estimates were very low for *A*. *lumbricoides* (0.8% and 0.2%), *T*. *trichiura* (0.6% and 0.8%) and *S*. *mansoni* (1.3% and 1.8%), in annual and semi-annual MDA areas, respectively. Therefore, we focused on hookworm for all further analyses.

### Impact of MDA using combined ivermectin and albendazole on hookworm

**Fig 2:**
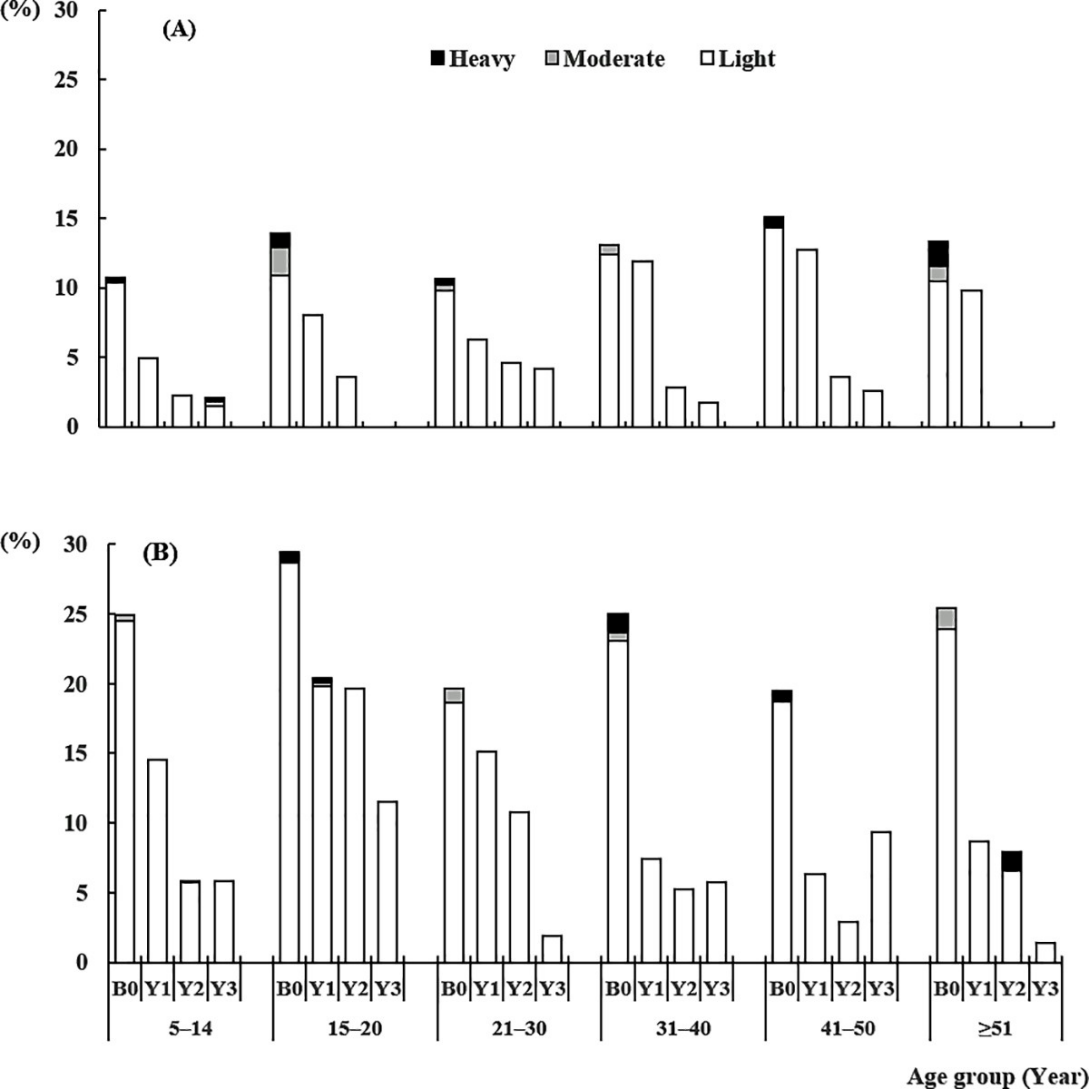
Hookworm infection intensity stratified by age group in sentinel areas of an MDA program to eliminate LF and onchocerciasis in eastern Côte d’Ivoire. (A), Akoupé health District with semi-annual MDA. (B), Abengourou health District with annual MDA. The last re-examination was performed 12 months after the last round of MDA. B0: Baseline, Y1: Year 1, Y2: Year 2, Y3: Year 3.

Hookworm prevalence and intensity data are provided in Table 1. Analysis by age group revealed a similar pattern of hookworm infection in both study areas, with low prevalence and light intensity among children under 10 years, and higher prevalence and heavier intensity in older participants (Fig 2). At baseline, the prevalence of moderate and heavy hookworm infections was 1.3% and 1.0% in the semi-annual and annual treatment areas, respectively, and dropped to 0.4% and 0.0% at year 3. The prevalence of heavy and moderate hookworm infections was generally low, and only single cases were detected, especially at baseline (Fig 2). Over three years of the MDA program, the infection was more rapidly reduced in adult subjects from both annual and semi-annual MDA areas. Reduction of mean intensity of hookworm infection was higher in females in the semiannual MDA area, but reduction of hookworm prevalence was higher in males in the annual MDA area (Table 2).

**Table 1:** Crude prevalence and intensity levels of hookworm infection in annual (1X) and semi-annual MDA area (2X) from baseline to year 3.

Epidemiological parameter	Baseline	1^st^ follow up	2^nd^ follow up	3^rd^ follow up	Reduction
**Abengourou (1X)**					
Prevalence % (95% CI)	23.9* (21.3–26.5)	13.2 (11.2–15.2)	7.3 (5.5–9.2)	5.5* (3.5–7.4)	77
Arithmetic mean epg (95% CI)	406.2** (306.3–506.2)	260.4 (169.7–351.1)	305.2 (81.2–529.3)	118.3** (77.9–158.8)	70.9
Level of intensity					
No n (%)	784 (76.12%)	965 (86.78%)	693 (92.65%)	502 (94.54%)	
Light n (%)	236 (22.8%)	145 (13.04%)	53 (7.08%)	29 (5.46%)	
Moderate n (%)	6 (0.6%)	1 (0.90%)	0 (0.0%)	0 (0.0%)	
Heavy n (%)	4 (0.4%)	1 (0.90%)	2 (0.27%)	0 (0.0%)	
Total N (%)	1030 (100%)	1112 (100%)	748 (100%)	531 (100%)	
**Akoupé (2X)**	** **	** **	** **	** **	** **
Prevalence % (95% CI)	12.4* (10.3–14.4)	7.5 (5.7–9.3)	2.6 (1.4–3.8)	1.9* (0.8–3.0)	85
Arithmetic mean epg (95% CI)	804.9*** (516.1–1093.7)	135.0 (80.4–189.6)	136.0 (82.7–189.3)	875.0*** (0.0–2064.0)	−8.7
Level of intensity					
No n (%)	869 (87.6%)	739 (92.5%)	665 (97.1%)	618 (98.1%)	
Light n (%)	110 (11.1%)	60 (7.5%)	18 (2.9%)	10 (1.5%)	
Moderate n (%)	6 (0.6%)	0 (0.0%)	0 (0.0%)	1 (0.2%	
Heavy n (%)	7 (0.7%)	0 (0.0%)	0 (0.0%)	1 (0.2%)	
Total N (%)	992 (100%)	799 (100%)	683 (100%)	630 (100%)	

CI, Confidence interval

N, Number of individuals analyzed

n, Number of individuals with a state of infection

*, Baseline and 3rd follow up prevalences statistically different

**, Baseline and 3rd follow up intensity statistically different

***, Baseline and 3rd follow up intensity comparable

**Table 2:** Comparison of hookworm prevalence, mean intensity and intensity classes in infected male and female subjects by treatment area and year.

Parameter			Baseline					Year 1					Year 2					Year 3				
Health District	Age (Year)	Gender	n (%)	AM (epg)	L	M	H	n (%)	AM (epg)	L	M	H	n (%)	AM (epg)	L	M	H	n (%)	AM (epg)	L	M	H
Akoupé	≤14	Male	13 (8.7)	329.5	8.7	0.0	0.0	7 (5.9)	219.4	5.9	0.0	0.0	4 (2.2)	151.7	2.2	0.0	0.0	1 (0.5)	12	0.5	0.0	0.0
Akoupé		Female	20 (12.7)	574.8	12.0	0.0	0.7	5 (4.0)	81.6	4.0	0.0	0.0	3 (2.3)	96.0	2.3	0.0	0.0	6 (4.3)	1622.0	2.9	0.7	0.7
Akoupé	>14	Male	36 (14.2)	835.7	12.2	1.2	0.8	33 (11.5)	108.7	11.5	0.0	0.0	10 (5.3)	163.2	5.3	0.0	0.0	3 (1.6)	140.0	1.6	0.0	0.0
Akoupé		Female	54 (12.5)	984.0	10.9	0.7	0.9	15 (5.6)	171.2	5.6	0.0	0.0	1 (0.6)	36	0.6	0.0	0.0	2 (1.7)	168.0	1.7	0.0	0.0
Abengourou	≤14	Male	46 (30.9)	240.3	30.2	0.7	0.0	40 (20.9)	205.2	20.9	0.0	0.0	15 (7.5)	451.2	7.0	0.0	0.5	14 (8.3)	93.4	8.3	0.0	0.0
Abengourou		Female	21 (17.5)	209.7	17.5	0.0	0.0	17 (8.4)	346.6	8.4	0.0	0.0	7 (3.9)	222.9	3.9	0.0	0.0	3 (2.4)	232.0	2.4	0.0	0.0
Abengourou	>14	Male	90 (24.3)	414.5	23.8	0.0	0.5	55 (15.1)	303.0	14.5	0.3	0.3	24 (10.8)	121.5	10.8	0.0	0.0	9 (6.3)	100.0	6.3	0.0	0.0
Abengourou		Female	89 (22.8)	530.0	21.0	1.3	0.5	35 (9.9)	214.6	9.9	0.0	0.0	9 (6.1)	616.0	5.4	0.0	0.7	3 (3.1)	176.0	3.1	0.0	0.0

N, Number of individuals tested by Kato-Katz

n, Number of infected individuals

L, Light infection

M, Moderate infection

H, Heavy infection

epg, egg per gram of faeces

AM, Arithmetic Mean

### Comparison of annual and semi-annual MDA

The crude prevalence of hookworm in the annual MDA area decreased significantly from 23.9% at baseline to 5.5% at year 3 (*p* <0.001). In the semi-annual MDA area, the crude prevalence of hookworm fell also consistently from 12.4% at baseline (12.4%) to 1.9% at year 3 (*p* <0.001). The rate of decrease was comparable in both treatment areas. Over the three years of the study, the mean intensity of hookworm eggs in infected subjects was reduced to about a quarter (from 406.2 epg to 118.3 epg) in the annual MDA area (*p* = 0.017). In the semi-annual treatment area with lower prevalence of hookworm eggs at baseline, the intensity in infected subjects did not change significantly (804.9 epg vs 875.0 epg, *p* = 0.216) because of the low number of infected individuals.

Mixed logistic regression and mixed linear models were used for further analysis of hookworm presence and intensity. Results indicated that there were significant differences between treatments, and that these differences varied according to follow-up visit (Treatment x Visit, *p* = 0.018). Odds-ratio estimates indicated that the annual treatment had greater odds of hookworm presence at all of the follow-up visits (Table 3). However, the annual treatment area also had a higher baseline prevalence than the semi-annual treatment area (model adjusted prevalence in Abengourou: 22.2% [95% CI: 13.3, 34.7], Akoupé: 9.2% [95% CI: 5.3, 15.3]) indicating that differences between treatment groups may be driven by higher initial prevalence in Abengourou. In fact, after adjusting for baseline prevalence, only the year 2 follow-up visit indicated a difference between treatments (model adjusted odds ratio: 2.26 [95% CI: 1.03, 4.98], *p* = 0.043; Table 3).

**Table 3:** Mixed logistic regression analysis for annual (Abengourou) vs semi-annual (Akoupé) treatment comparisons at each follow-up visit after model adjustment for baseline prevalence. The odds ratio (OR) of the semi-annual treatment group was calculated relative to the annual treatment group.

	Not adjusted for baseline prevalence		Adjusted for baseline prevalence	
Visit	OR (95% CI)	p	OR (95% CI)	p
Year 1	3.65 (1.51, 8.81)	0.004	1.06 (0.53, 2.12)	0.871
Year 2	7.58 (2.79, 20.57)	< 0.001	2.26 (1.03, 4.98)	0.043
Year 3	5.87 (2.03, 16.94)	0.001	2.0à (0.81, 4.9)	0.131

OR, Model-adjusted odds ratio

CI, Confidence interval

*p*, P-value

Our analysis of hookworm intensity also indicated that treatment differences varied by follow-up visit (Treatment x Visit, *p* = 0.002). In contrast to the prevalence results, Akoupé had a higher mean intensity (adjusted geometric mean: 276.9 epg [95% CI: 216.9, 354.2]) than Abengourou (adjusted geometric mean: 145.0 epg [95% CI: 121.7, 172.7]; *p* = 0.054) at baseline. However, the number of infected subjects was lower in Akoupé and the confidence interval was larger. In the first follow-up visit, Abengourou had higher hookworm intensity (adjusted geometric means: 101.9 epg [95% CI: 82.6, 125.8] vs 65.1 epg [95% CI: 48.4, 87.6], *p* = 0.029), but the mean intensities between treatment areas were not significantly different in subsequent follow-up visits (*p* = 0.871 for year 2 and *p* = 0.258 for year 3).

## Discussion

A goal of the WHO is to control STH and reduce the prevalence of moderate and heavy infections below 1%. This may eliminate STH infections as a public health problem and possibly break transmission [26]. A key tool to achieve this goal is preventive chemotherapy. In the current study, LF and onchocerciasis are co-endemic in the study areas and a combination of IVM with ALB is used for MDA as part of the national programs to eliminate LF and onchocerciasis. IVM and ALB is one of the most efficacious drug combinations for hookworm with average cure rates of 84% and egg reduction rates of 95% [27]. Therefore, IVM plus ALB provided for LF and onchocerciasis elimination should also reduce STH, but little field data have been published to quantify this effect on the community level [15]. Indeed, this may be the first community-based study that measured the impact of annual and semi-annual MDA for LF on STH in West Africa.

In our study, the self-reported compliance recalled during the next survey 6 to 12 months after MDA in both treatment areas was sometimes lower than the 65% target for LF elimination programs [21]. However, an independent coverage survey [C. Comoe-Coffie, *pers*. *comm*.] using a standardized questionnaire [23] documented a compliance rate of 71.3% in our semi-annual area. It is possible that a proportion of subjects questioned in the independent survey may have confused administration of IVM and ALB with another type of drug swallowed. However, it is more likely that the low compliance rate in our survey was caused by a lack of accurate recall 6 to 12 months after MDA. A study performed in nine communities in Kebbi State, Nigeria indicated that it is difficult to achieve 65% compliance during annual MDA with IVM and ALB [28]. However, studies from other regions showed that it is feasible to achieve high compliance rates over several rounds of MDA [29]. High compliance rates are crucial for successful intervention and previous studies identified a number of challenges that vary widely between settings and rounds of MDA [30–32].

Our results showed that MDA using IVM combined with ALB to eliminate LF and onchocerciasis had a strong impact on hookworm prevalence and intensity. While drug efficacy is usually measured 2 to 4 weeks after treatment in a cohort, the impact of MDA on STH in this study was assessed one year following treatment in a cross-sectional approach. After three years of community-wide MDA, hookworm prevalence dropped from 23.9% to 5.5% and from 12.4% to 1.9%, with reductions of 77% and 85% in the annual and semi-annual treatment areas, respectively. These results are in line with cure rates of about 80% determined by meta-analysis of a number of clinical trials [27, 33, 34]. Although several computer modeling studies predict the potential impact of community-wide MDA on hookworm prevalence [35, 36], field observations from Africa are scarce. Our logistic regression analysis results indicated that, after adjusting for differences in baseline prevalence, we were able to detect a difference between both treatment regimens at only one of the three follow-up visits. However, it has to be taken into account that only the Year 3 follow-up visit was done 12 months after the last MDA round for both treatment groups, while in Year 1 and 2, evaluation was performed 12 months after the last MDA in the annual MDA group and 6 months after the last MDA in the semi-annual MDA group.

Our results suggest that differences in hookworm prevalence reduction between the treatment areas are primarily driven by geographic differences and baseline prevalence. The similar effects of the different treatment regimens (5 vs 3 rounds of MDA) may be explained by the life cycle of the parasite. Annual MDA may be sufficient to eliminate most hookworms as infective larvae, adult worms, and eggs because the drugs are larvicidal, vermicidal, and ovicidal. Hookworm infection is caused by the penetration of infectious, free-living L3 larvae that survive only a few months in the soil [37]. Moreover, the study was done in low and moderate endemic settings. Therefore, the infection pressure may be relatively low and re-infections may be efficiently prevented even with annual community-wide MDA. Semi-annual and annual MDA reduced the prevalence of moderate and heavy infections to 0.4% and 0%, respectively. Thus, the appropriate threshold (<1%) for successful control of hookworm as defined by WHO was achieved by both MDA regimens. During the third follow-up, relatively few subjects were hookworm positive, but some of them had a relatively high intensity of infection. This may be an indicator of non-compliance [38] rather than an indicator of heavy transmission in the study villages and may call for specifically targeting groups of subjects who more likely to be non-compliant with MDA [39].

The present study had two principal limitations. The reported compliance with MDA was sometimes lower than the WHO recommended coverage level (65% for LF elimination and 75% for STH control). This may impact the proper comparison between annual and semi-annual MDA. The second limitation is that our cross-sectional study design did not randomly select the villages within an health District, because selection of study villages was guided by LF and onchocerciasis prevalence. It has to be considered that we did not repeat STH testing of infected individuals 2–4 weeks after treatment to assess cure and egg reduction rates. Barriers that could prevent individuals to be continuously included in parasitological survey would be the absence from the village, misunderstanding, and lack of information [40]. However, our study was not designed to assess drug efficacy, but to measure impact of an intervention (MDA for LF and onchocerciasis), and cross-sectional sampling was sufficient for this purpose.

In conclusion, both annual and semi-annual community-wide MDA for LF elimination with IVM and ALB over three years resulted in dramatic reductions in hookworm prevalence in low and moderate endemic settings in eastern Côte d’Ivoire. Thus, annual MDA for LF elimination appears sufficient for effective hookworm control in areas with low to moderate baseline prevalence, and separate school-based deworming is not necessary in such areas.

## Supporting information

S1 Checklist STROBE checklist for cross-sectional studies. (DOC)

S1 Table Coordinates of the study villages depicted in Fig 1. (XLSX)
